# Editorial

**Published:** 1841-07-17

**Authors:** 


### THE MEDICAL EXAMINER.

PHILADELPHIA, JULY 17, 1841.

### MEDICAL REFORM IN THE UNITED STATES.

The great principle of medical reform now agitated in Great Britain—a uniform system of examinations for degrees—has been adopted by the profession in this city, who united in a nearly unanimous effort to establish the Philadelphia College of Medicine. This step, we believe, met with the general sanction of such of the profession throughout the country as took an interest in the subject. The object in view, was to erect a board of examiners for the qualification of practitioners disconnected with the business of instruction, which was to be thrown open to all competitors. When the Philadelphia College of Medicine was organized, we expressed a doubt whether "it would go into extensive operation so long as the other faculties of medicine retained the power to grant degrees."* We thought it improbable that the students who attended the lectures of the chartered institutions would resort elsewhere for degrees, while new combinations of teachers would be unwilling to start without the privilege enjoyed by those already in the field. The resnlt has borne out these views. The College of Medicine, with an excellent organization, has done nothing since its creation; while a third medical faculty has been added to the two older institutions, and has found no difficulty in obtaining a charter with the usual privilege. It is therefore apparent that the desired reform in medical education is unattainable without a surrender on the part of

* See Medical Examiner, vol. 2d, No. 44.

the existing institutions of their special privileges. The questions then arise:—ought such a surrender to be asked for? Can it be obtained? Can a uniform system be established in its place?

Before discussing the two former questions, a satisfactory answer must be had for the last. The clear prospect of something better must be shown before we attempt to disturb a system of machinery which has confessedly worked well. The subject is by no means free from difficulty. Much is encountered in the attempt to apply a uniform system to a country like Great Britain, of small area, densely populated, with an all powerful central government. The difficulty will of course be enhanced in our own vast continent, with its scattered population and conflicting State governments. How far it may be surmounted is a matter for careful examination, which we propose to enter upon in our next number. With the conviction that the reform in view is of vast importance, we shall not be turned aside in the effort to bring it about by the apparent magnitude of the obstacles encountered. That these may be overcome, we think, and hope to be able to show.

---

The prevalent disorders of the season at Philadelphia, as in most cities of the United States, are the inflammatory affections of the alimentary canal. These vary in each year. Thus, in the year 1838, the bowel affections were extremely severe, and passed rapidly into the malignant or sloughing form. In 1839 and 1840 they were much more inflammatory, and generally yielded readily to treatment. In the present season the cases are of a mixed character, much less inflammatory on the whole, however, than those of last year.

The treatment by calomel, ipecac. and opium, pushed to ptyalism, which acted as a specific the last year and in previous years, has not been quite so successful, that is, ptyalism was not accompanied, in a great proportion of cases, with the very decided relief which usually follows in the inflammatory variety.

Oily mixtures, with the addition of small portions of blue mass, have been more useful in our hands than any single remedy in the present epidemic; but the disease has presented the usual diversity in symptoms and treatment.

